# Dermal Fibroblasts as the Main Target for Skin Anti-Age Correction Using a Combination of Regenerative Medicine Methods

**DOI:** 10.3390/cimb45050247

**Published:** 2023-05-01

**Authors:** Alla Zorina, Vadim Zorin, Artur Isaev, Dmitry Kudlay, Maria Vasileva, Pavel Kopnin

**Affiliations:** 1 The Human Stem Cells Institute, Moscow 119333, Russia; 2 SKINCELL LLC, Moscow 119333, Russia; 3 Department of Pharmacology, The I.M. Sechenov First Moscow State Medical University (The Sechenov University), Moscow 119991, Russia; 4 The N.N. Blokhin National Medical Research Oncology Center, The Ministry of Health of Russia, Moscow 115478, Russia

**Keywords:** skin structure, skin fibroblasts, laser therapy, correction of age-related skin changes, cell therapy, PRP therapy

## Abstract

This article includes the data from current studies regarding the pathophysiological mechanisms of skin aging and the regenerative processes occurring in the epidermis and dermis at the molecular and cellular level, mainly, the key role of dermal fibroblasts in skin regeneration. Analyzing these data, the authors proposed the concept of skin anti-age therapy that is based on the correction of age-related skin changes by stimulating regenerative processes at the molecular and cellular level. The main target of the skin anti-age therapy is dermal fibroblasts (DFs). A variant of the cosmetological anti-age program using the combination of laser and cellular methods of regenerative medicine is presented in the paper. The program includes three stages of implementation and defines the tasks and methods of each stage. Thus, laser technologies allow one to remodel the collagen matrix and create favorable conditions for DFs functions, whereas the cultivated autologous dermal fibroblasts replenish the pool of mature DFs decreasing with age and are responsible for the synthesis of components of the dermal extracellular matrix. Finally, the use of autological platelet-rich plasma (PRP) enables to maintenance of the achieved results by stimulating DF function. It has been shown that growth factors/cytokines contained in α-granules of platelets injected into the skin bind to the corresponding transmembrane receptors on the surface of DFs and stimulate their synthetic activity. Thus, the consecutive, step-by-step application of the described methods of regenerative medicine amplifies the effect on the molecular and cellular aging processes and thereby allows one to optimize and prolong the clinical results of skin rejuvenation.

## 1. Introduction

The skin is the largest multifunctional human organ and possesses a complex multi-layered structure with the ability to regenerate and renew. All layers of the skin have morphofunctional integrity, and each layer has a specific function, structure, and cellular composition, which correlatively change throughout a person’s life [1,2,3,4,5,6].

Both chronological aging and photoaging are accompanied by a decrease in skin function, mainly regenerative, and, therefore, a decrease in the skin’s ability to renew and maintain proper homeostasis. The skin involution is primarily based on the changes associated with the main cell population of the skin, dermal fibroblasts (DFs), namely, their number and functional activity (see review Zorina et al., 2022 [4]). DFs play a key role in skin biology. DFs support homeostasis of the dermal extracellular matrix (ECM), providing its remodeling, which, in turn, creates favorable conditions for DF functioning. DFs play an important role in maintaining the physiological status of the other skin layers (see review Zorina et al., 2022 [5]). The changes in the population of DFs correlate with structural, compositional, quantitative, and qualitative transformations of the main ECM proteins in the dermis, as well as with the age-related disorders of epidermal morphogenesis and the state and proliferative activity of basal keratinocytes (BKs) that are the main cells in the epidermis [6,7]. This suggests that the effect of anti-age skin therapy directly depends on the stimulation of DF activity and the increase in the number of DFs.

Obviously, the use of regenerative medicine methods, in particular, laser technologies, cell therapy methods, etc., as a part of anti-age skin correction programs is expedient and relevant since the methods enable the activation of DF functions. We believe that the complex (combined) application of these methods allows one to achieve the greatest effect. Thus, the laser treatment causes thermal damage to the epidermis and the upper layer of the dermis, which promotes the reparative processes in these skin layers; namely, the epidermis is renewed, and the collagen matrix is remodeled, which in turn stimulates DF functions and, therefore, skin revitalization. In combination with the laser treatment, the use of cellular technologies, in particular, LAVIV^TM^: http://dokubiyoteknoloji.com/en/fibrocell (accessed on 23 April 2023) or SPRS^®^: http://sprs-therapy.com (accessed on 23 April 2023) therapies, has been shown to be beneficial. Such therapies based on the introduction of cultivated autological DFs (autoDFs) into the skin is aimed at increasing the number of functionally active DFs and stimulating resident DFs. In addition, PRP therapy has also been shown to be useful since it stimulates DFs in the epidermis and dermis, simultaneously potentiating the cumulative effect of laser exposure and cell therapy. This approach, taking into account the biological mechanisms that are individual for each person, enables improving the health, function, state, and quality of the skin, thereby achieving a more youthful skin appearance and, moreover, maintaining the resulting clinical effect for a long time. Accordingly, the development and application of the proposed combination of regenerative methods require specialists to have a deep understanding of the pathophysiology of skin aging and the mechanisms of the regenerative processes occurring in the epidermis and dermis at the molecular and cellular levels. It is generally important to consider the key role of dermal fibroblasts in skin regeneration processes.

A brief review is provided within the framework of this article concerning two layers of the skin, the epidermis and the dermis, since they are the main targets for the cosmetological anti-age program, which is the focus of the present work.

## 2. Skin Cells and ECM Components Playing a Key Role in Skin Regeneration

### 2.1. Keratinocytes as the Main Cells of the Epidermis

**Figure 1:**
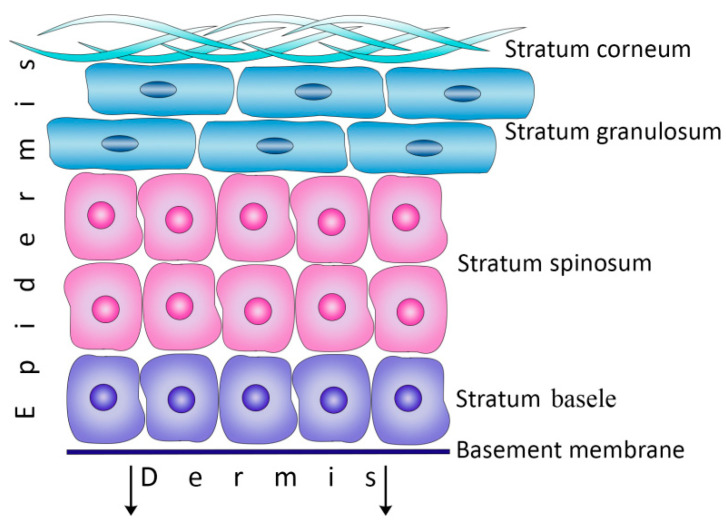
The structure of the epidermis.

The interfollicular epidermis is the outer skin layer that protects the body from external influences and dehydration, as well as determines the texture, moisture, and color of the skin [1,2,8]. The epidermis represents the constantly renewing epithelial tissue possessing high regenerative properties and consisting of four main layers: basal (*stratum basal*), spinous (*stratum spinosum*), granular (*stratum granulosum*), and cornified (*stratum corneum*) layers (Figure 1). Epidermal homeostasis is maintained due to the proliferation and differentiation of keratinocytes that are located in the basal layer (basal keratinocytes [BKs]) and attached to the underlying basement membrane (BM) through the transmembrane integrin heterodimers synthesized by BKs. The integrin heterodimers are connected with the ECM ligands located in the basement membrane to provide communication with the cellular cytoskeleton [9,10,11].

Two populations of proliferating cells were distinguished among epidermal BKs: epidermal stem cells (ESCs) and transit-amplifying cells (TACs). ESCs, making up to 10% of BKs, are characterized by high mitotic activity, while TACs, being a daughter generation of ESCs, are the committed progenitor cells [12]. As soon as TACs lose their connection with the BM, they exit the cell cycle and begin the program of final differentiation into keratinocytes, which subsequently form the upper layers of the epidermis [9] (Figure 1). 

### 2.2. Dermal Fibroblasts as a Key Link in Skin Biology

The dermis is located under the epidermis and represents the main skin layer, which plays a key role in the proper functioning of the skin. The transition from the epidermis to the dermis is provided by the dermal–epidermal junctions (DEJs), which are complex formations. The main component of DEJs is the basement membrane (BM), which includes the following proteins: laminin 1, laminin 5, nidogen, and type IV collagen (basement membrane collagen), forming the network [1]. The BM serves as a mechanical support for cells, and it also regulates the removal of cellular metabolism products and the intake of nutrients and the growth factors/cytokines produced by DFs and, therefore, plays a significant role in the regulation of epidermal morphogenesis [13,14]. Directly beneath the epidermis, the network of “anchor” collagen fibers of type VII is located and introduced into the BM. The network stabilizes the skin structure by strengthening the connection between the epidermis and the underlying papillary dermis, thereby ensuring the tight fit of the BM to the papillary dermis [8]. Directly under the BM, immature oxytalan elastic fibers smoothly turn into elaunin fibers and collagen fibers of type III. The complex network, consisting of the interconnected BM proteins and papillary proteins of the dermis, contributes to the structural integrity and mechanical stability of the skin [15].

**Figure 2:**
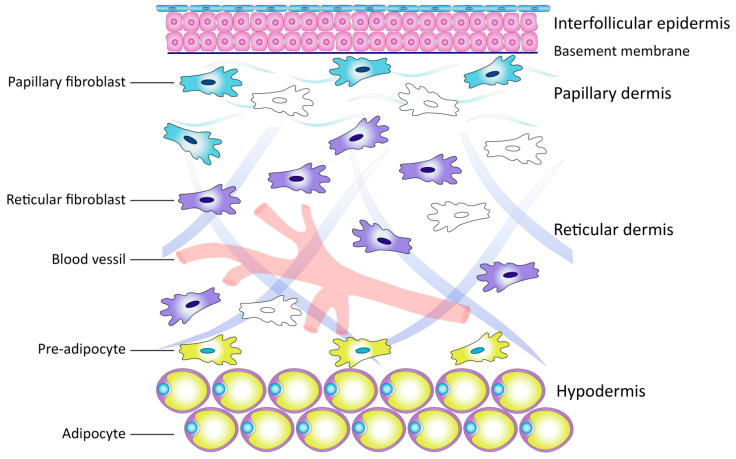
Schematic representation of the skin and localization of cells.

The dermis represents the framework consisting of the ECM and many types of cells, namely: pericytes, endothelial cells, smooth muscle cells, immune cells, and fibroblasts. Each cell type performs a specific role (Figure 2) [1,13]. Within this cellular diversity, fibroblasts are the main cells, providing homeostasis and morphofunctional organization of the skin. According to M. Sorrel and A. Kaplan (2009), these cells are the key link in the biology of the skin [13].

Dermal fibroblasts (DFs) control the structure and composition of the dermal ECM, including collagen, elastin, proteoglycans, and structural glycoproteins. The DFs functions include not only the production of ECM substances but also their catabolism by means of the secretion of collagenases, cathepsins, and hyaluronidases, as well as by the direct phagocytosis of fibrils [13].

The stroma formed by fibroblasts serves as the framework that physically supports the epithelium and also regulates the structure/functions of the epithelial cells and epidermal morphogenesis [9,13,14,15]. DFs produce factors that regulate the proliferation and differentiation of keratinocytes, including keratinocyte growth factors 1 and 10 (KGF-1 and KGF-10, also known as KGF-2), granulocyte-macrophage colony-stimulating factor (GM-CSF), epidermal growth factor (EGF), interleukin 6 (IL-6), etc. [16,17,18,19]. Researchers believe that the status of epidermal tissue is determined not only by the paracrine effect of these factors but also by their ratio; in particular, the ratio of KGF-1 to GM-CSF plays a significant role in this process [20]. Interleukin 1 (IL-1), produced by keratinocytes, regulates these factors, indicating the importance of intercellular relationships in maintaining the homeostasis of skin tissues [19]. This was confirmed by Philippeos et al., 2018. The study has revealed the synergetic and reciprocal interactions mediated by the Wnt signaling pathway between DFs located in the papillary layer of the dermis and basal keratinocytes; moreover, the pathway maintains the cellular identity of DFs [21]. In the experiments conducted in vitro, the elimination of this signaling pathway can explain the rapid disappearance of markers characteristic of DFs in the papillary dermal layer. In addition, DFs play a significant role in establishing the structure of the BM by producing collagens of types IV and VII, laminin 1, and entactin/nidogen [13,22,23].

DFs secrete the factors affecting lymphocyte differentiation and also the factors that regulate the number of granulocytes and macrophages and their migration and functions, thereby maintaining skin immunity [1,3,24]. DFs are actively involved in angiogenesis through the production of many proangiogenic factors (VEGFs, FGFs, TGF-β1, HGF/SF, and angiopoietin 1) that induce the differentiation and migration of endothelial cells and contribute to the formation and stabilization of blood vessels [8,15]. Studies have also shown that DFs are involved in the processes of the neuroendocrine regulation of skin functions. DFs produce biologically active peptides (hormones, biogenic amines, neuropeptides, and neurotransmitters) that are identical to those synthesized by the central nervous and endocrine systems. DFs also synthesize prolactin, which is identical to the hypothalamic one, and produce the sex hormone 17-beta-estradiol. DFs express androgen and estrogen receptors mediating the effect of these hormones on human skin [15].

Dermal fibroblasts represent the heterogeneous cell population, including cells of the same cytogenetic line characterized by different degrees of differentiation and specific properties determined by the fibroblastic differon [13,24,25,26,27,28,29,30]. The fibroblastic differon includes:

- fibroblast progenitor cells (mesenchymal stem (stromal) and progenitor cells) possessing high proliferative potential and maintaining the number of fibroblasts in the dermis;
- mature (differentiated) and postmitotic fibroblasts make up the majority of cells in the dermis; they no longer divide in the skin in vivo but have high biosynthetic activity, producing and organizing all ECM components, which determines their main role in the fibroblastic differon;
- specialized fibroblasts (such as fibroblasts that resorb ECM, myofibroblasts that possess contractility, and fibrocytes), which are represented by the finally differentiated cells having minimal producing activity and maintaining cellular homeostasis in the skin.

In addition, the skin contains populations of specialized fibroblasts associated with establishing the structure of hair follicles [1,9].

#### Subpopulations of Dermal Fibroblasts

The dermis consists of two layers: the superficial papillary layer (making up about a third of the dermis) located directly under the epidermis and the lower reticular layer (making up the bulk of the dermis) [1,31]. The papillary layer consists of fibrous soft unformed connective tissue characterized by a high density of fibroblasts. The papillary layer possesses a dense mesh structure consisting of randomly arranged thin collagen fibers (mainly of type III) and immature (oxytalan and elaunin) fibers of the elastic network. The reticular layer consists of fibrous and dense unformed connective tissue and contains highly ordered thick bundles of collagen fibers (mainly of type I) and mature elastic fibers. The reticular layer is characterized by a low density of fibroblasts. These two dermal layers differ in volume, density, and DF composition, as well as in the composition and organization of the ECM. They contain different subpopulations of DFs possessing unique morphologies and physiological functions [13,21,24,25,26,27,28,32].

The study of transcription profiles conducted by Philippeos et al. (2018) has confirmed the occurrence in the dermis of two different fibroblast subpopulations with phenotypes that are specific to each dermal layer [21]: the subpopulation of papillary dermal fibroblasts (pDFs) with phenotype lin-CD90 + CD39 + CD26− and the subpopulation of reticular dermal fibroblasts (rDFs) with phenotype lin-CD90 + CD36+, which is also characteristic of hypodermal preadipocytes. The expression of the genes responsible for cytoskeleton structure and cell mobility mainly occurs in the rDF population, whereas the high expression of the genes responsible for the complement activation pathways prevails in the pDF population, which indicates that pDFs are involved in skin immune system activity [28]. It has also been shown that the pDF population differs from the rDF population in the larger proportion of fibroblast progenitor cells, possessing high proliferative potential, whereas the mature (differentiated) fibroblasts are mainly localized in the reticular layer [30].

### 2.3. Extracellular Matrix of the Dermis

The dermal ECM components synthesized by DFs can be divided into three main groups [31,32]. The first group includes the fiber-forming structural elements that support the ECM by creating the complex three-dimensional structure that determines the strength and elasticity of skin tissues. The most common fiber-forming protein in the skin is collagen, which makes up about 80% to 90% of the dry weight of the skin, while the other proteins are represented by fibrin, fibronectin, vitronectin, elastin, and fibrillin.

The second group includes structural ECM components that do not form fibers, mainly proteoglycans and glycosaminoglycans, whose function is to create the dynamic and osmotically active ECM environment for dermal cells and that also participate in cell adhesion and migration. They make up a large part of the interstitial space, while their negative charge and hydrophilic nature provide the hydration and physiological pH of skin tissues [31,32]. These ECM components are dispersed throughout the dermis and play a key role in maintaining skin hydration [33,34]. 

The third group of the ECM components of the dermis consists of specialized proteins (the family of intercellular signaling proteins (the CCN protein family) as well as growth factors, cytokines, and chemokines, etc.) that provide the interaction between DFs and ECM through auto- and paracrine signaling pathways [31,33,34].

#### 2.3.1. Collagen

Special attention should be paid to protein collagen, which forms the basis of the skin’s connective tissue. Collagen is represented in the dermis by a large family currently including 11 collagen types that significantly differ from each other [35,36,37,38,39,40,41,42,43,44,45] (Table 1).

The main framework of the dermis consists of fiber-forming (fibrillar) collagens. Type I collagens make up about 80% of all types of fibrillar collagens, whereas type III and type V collagens constitute ~15% and ~5%, correspondingly. Collagens of types I and III (large fiber-forming, classical, interstitial collagens) form the main structural network of the dermis [35,36,37]. Fibrillar collagens are located both in the papillary dermis (mainly type III collagen) and in the reticular dermis (type I collagen) [1,30]. Type V collagen (minor fiber-forming collagen) does not form collagen fibers by itself; however, it initiates the inclusion of type I and type III collagens into the fibers by occurring in the “core” of the fibers [38].

Non-fibrillar types of collagens also have a significant impact on the characteristics of the structural network. Thus, FACIT collagens (possessing an intermittent triple helix) include collagens of types XII, XIV, XVI, and XXII, which are located on the surface of fibers formed by large fibrillar collagens; thereby, they play an essential role in regulating the assembly of the collagen network and the interaction of these collagen fibers with the non-collagenic components of the ECM, namely, proteoglycans and elastic fibers [39,40]. In addition, collagens of types XII and XVI are mainly localized in the papillary dermis, while type XIV collagen is situated in the reticular dermis [37]. These types of collagens are believed to promote the interaction of type I and III collagen fibers with decorin and perlecan (from the BM proteoglycans family), which are involved in the regulation of fibrillogenesis [40,41].

Type VI collagen, which is widely distributed in most connective tissues of the body, belongs to the family of collagens that form thin microfibrils and whose shape resembles prayer beads. However, the role played by the microfibrils formed by this collagen type in the skin has not been fully clarified. They are believed to participate in the interaction between cells and the ECM [42,43]. DEJ collagens are mainly represented by collagens of types IV and VII, which provide a tight connection between the BM and the papillary layer of the dermis and, therefore, stabilize the skin structure by strengthening the linkage between the epidermis and the dermis [8].

#### 2.3.2. Elastic Network of the Dermis

The elastic network of the dermis is formed by fibers of three types: elastic fibers proper (mature fibers consisting of fibrillin and elastin), oxytalan fibers (immature fibers solely formed by fibrillin), and elaunin fibers (the intermediate form between oxytalan fibers and elastic fibers proper) [46]. Each layer of the dermis is characterized by a specific elastic network. The elastic fibers proper are characteristic of the reticular layer; most fibers are located parallel to the surface of the skin, whereas the rest of the fibers are disposed perpendicular to the surface of the skin and connect the parallel fibers. In the papillary layer of the dermis, the elastic network mainly consists of thin elaunin fibers located perpendicular to the epidermis. In the upper part of the papillary dermis, elaunin fibers are attached to the cascade of also perpendicularly located oxytalan fibers that are directly connected to the BM [31,32,46,47]. The features of the elastic system, in particular the fibrillin/elastin ratio, can serve as parameters that allow one to distinguish between the papillary and reticular layers of the dermis.

Thus, the dermis is the integrating basis of the skin. DFs are the main cells of the dermis that produce and remodel the dermal ECM components, secrete growth factors/cytokines that support epidermal morphogenesis and angiogenesis in all skin layers, participate in the formation of the BM, and maintain skin immunity; therefore, DFs are responsible for skin homeostasis as well as the morphofunctional organization and renewal of the skin [15,32,48,49]. By interacting with each other and with the other cells, DFs have a significant impact on the entire cellular community of the skin [10,15,30], playing a key role in skin regeneration.

## 3. Molecular and Cellular Changes in the Skin during the Aging Process

Both types of skin aging, chronological and photoaging, have specific clinical and morphological features [50,51]. Chronological aging is a genetically determined process that depends on the number of lived years, whereas photoaging directly depends on the degree of exposure to ultraviolet rays as well as the genetically predetermined degree of skin pigmentation [52,53,54]. Chronologically aged skin that has not been exposed to prolonged sunlight is characterized by thinning, decreased elasticity and firmness, pallor, and the occurrence of fine surface wrinkles. The symptoms of photoaging can be observed even before the appearance of signs of chronological aging and include the following signs: skin thickening, coarsening, dryness, deep wrinkles, telangiectasias, irregular pigmentation, and solar lentigo. In addition, the destructive changes occurring in the skin during photoaging are superimposed on the chronological aging processes and, thereby, accelerate their development [55].

With age, specific pathological changes occur in each layer of the skin.

However, especially significant changes occur in the dermis, and they are related to two types of fundamental molecular mechanisms (see reviews [4,5]). One of the mechanisms is related to the number and functional activity of DFs, which are the main cells in the dermis [56,57]; the other one is connected to the disturbed homeostasis of the dermal ECM and its main structural component, collagen [57,58,59].

In the process of aging, the number of DFs decreases (on average, the total number of DFs is 35% less in elderly people compared with young people [57]) primarily due to the depletion of fibroblast progenitor cells, which, in turn, inevitably leads to a decrease in differentiated (mature) fibroblasts, which are responsible for the production and organization of the ECM’s dermal components [60,61,62,63]. Cellular aging plays an essential role in this process and is accompanied by the formation of senescent fibroblasts (senDFs) that accumulate in the dermis with age [64,65]. SenDFs (like other senescent cells) are characterized by irreversible cell cycle arrest as well as by their resistance to apoptosis and the appearance of the Senescence-Associated Secretory Phenotype (SASP) [66,67]. SASP includes many proinflammatory cytokines/chemokines, growth factors, matrix metalloproteinases (MMPs), and other soluble factors [66,68]. Due to the secretion of these factors, senDFs contribute to the aging of neighboring stem cells through the paracrine pathway; they also cause the inflammation of dermal tissues (inflammaging) and induce the destruction/degradation of the dermal ECM through the action of MMPs. All of these processes that occur with age in the fibroblastic differon lead to impaired homeostasis of the skin’s connective tissues and, therefore, decrease the regenerative potential of the skin.

In addition, skin aging is accompanied by structural, qualitative, and functional disturbances in the dermal ECM that are associated with the ultrastructural, morphometric, mechanical, and conformational changes caused mainly by the impairment of collagen homeostasis [57,59,69,70,71,72]. These changes are based on molecular mechanisms activating the transcription factor AP-1 (activating protein 1) in DFs. AP-1 plays a central role in collagen destruction and represents a key link in the pathogenesis of both types of skin aging [53]. AP-1 promotes the expression of genes encoding matrix metalloproteinases (MMP-1, MMP-3, and MMP-9), which play a major role in the degradation of the ECM [73,74]. This results in the fragmentation and destruction of dermal collagen [75]. In addition, the activation of AP-1 is accompanied by a decrease in collagen production [53]. Thus, it has been revealed that in the DFs of elderly people compared with young people, a significant decrease occurs in the expression of the genes responsible for collagen production, and, by contrast, there is an increase in the expression of genes responsible for the production of MMPs. During photoaging, all of these processes are even more intensified due to the direct activation of AP-1 by ultraviolet rays [76]. As a result, destructive changes in the collagen matrix develop with age, namely a decrease in collagen production and an increase in the content of fragmented collagen [53]. Collagen fibers become significantly more rigid and randomly oriented; the irreversible modification of collagen occurs due to the formation of new cross-links, which slows down its turnover [72,77,78]. Such crosslinks are represented by the advanced glycosylation end products (AGEs) that accumulate in the ECM with aging.

**Figure 3:**
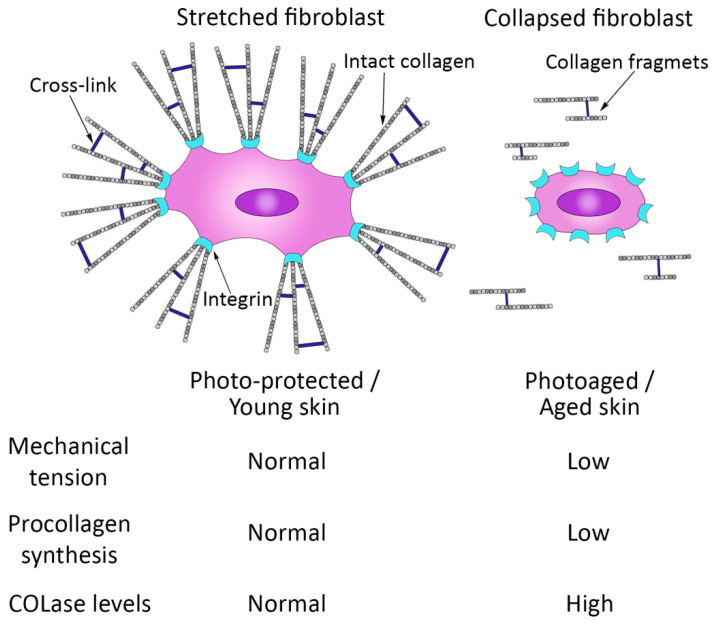
Schematic representation of the relationship between mechanical tension and collagen production and fragmentation in human skin.

The state of the collagen matrix, in turn, has a significant impact on DF functions [53]. The impairment of its integrity disrupts the focal contacts between the ECM and DFs, which leads to DFs losing their stretched state, which is characteristic of these skin cells at a younger age (Figure 3). A kind of fibroblast collapse occurs, which is accompanied by a disruption of their functions; in particular, there is a suppression of collagen synthesis and an increase in the production of MMPs [55,57]. A vicious circle forms, contributing to the progression of skin aging [57].

Thus, the disorders observed during skin aging, mainly the decrease in the skin’s ability to regenerate and renew, are directly caused by the age-related changes in the state and functions of DFs, the main cellular component of the dermis, and also by changes in the ECM produced by DFs. Taking into account the key role of DFs in skin regeneration processes, as well as in the processes of skin aging, it seems reasonable to use a combination of regenerative medicine methods for skin anti-age correction since this allows one to affect this cell population specifically.

## 4. A Variant of the Skin Anti-Age Correction Program Using a Combination of Laser and Cellular Methods

The purpose of the program is to correct age-related skin changes taking into account the fundamental mechanisms of aging and the regeneration of skin tissues. The skin anti-age correction program includes a combination of regenerative medicine methods (laser, cellular therapy, and PRP) and consists of three stages.

### 4.1. Program Stages: The Solved Tasks and Recommended Methods

#### 4.1.1. The First Stage

The objectives of the first stage of the program are to remodel the ECM of the dermis and, thereby, create favorable conditions for the functioning of DFs. Currently, there are many methods using medical devices, injections, and local preparations that allow one to remodel the dermal collagen matrix and improve the state of the epidermis [52,53,79]. The use of topical retinoids (vitamin A derivatives) can serve as an example. The retinoids act through nuclear retinoic acid receptors (RARs), which have two binding sites: one for the retinoid ligands and one for the specific DNA sequences of the target gene [80]. Thus, retinoids regulate the expression of the genes responsible for the synthesis of MMPs and collagen, thereby contributing to a decrease in the production of MMPs and an increase in the production of collagens of types I, III, IV, and VII [53,79,81,82]. This method is quite effective in correcting the manifestations of skin aging (primarily, photoaging); however, in order to obtain the desired clinical effect, it is necessary to conduct retinoid therapy for a long time (several weeks) [79,80], which is not always convenient for the patient. For this reason, from our point of view, the use of laser technologies is preferable to conduct the first stage of the anti-age program.

##### Dermal ECM Remodeling Using Laser Technologies

The mechanisms of the laser’s effect on skin tissues are explained by the theory of selective photothermolysis [83]. Among laser technologies, the gold standard is the ablative skin resurfacing technique using a carbon dioxide (CO_2_) laser. The CO_2_ laser causes thermal damage, leading to the ablation of the epidermis and also affects the papillary layer of the dermis [84]. The response of skin tissues to injury occurs through the activation of a highly organized cascade of molecular and cellular mechanisms that are similar to the mechanisms that participate in the physiological reaction during wound healing [83,85]. This process results I the restoration of skin tissues with the consequent production of collagen and the remodeling of the dermal ECM. The process of skin restoration after thermal damage includes three phases. The first (inflammatory) phase begins with a rapid increase in the level of pro-inflammatory cytokines IL-1β and TNF-α, which induces the expression of transcription factor AP-1 (activating protein 1) [84,86]. This leads to an increase in the level of MMP-1, MMP-3, and MMP-9, which are required for the degradation of the ECM and the purification of the dermis of the damaged ECM components. The second (reparative) phase is accompanied by the migration of DFs to the damaged skin zones with their subsequent proliferation and differentiation into reparative DFs, which intensively synthesize collagen and other ECM components of the dermis to completely repair the thermal damage [85,86]. At the same time, there is an increase in the level of transforming growth factor (TGF-β), which regulates the expression of genes encoding fibrillar collagens of types I and III [83]. This results in the formation and deposition of new collagen [85,86]. In addition, after the CO_2_ laser treatment, an increase in the levels of tropoelastin and fibrillin 1, the main components of elastic fibers, is also observed [85].

The essential difference between the tissue healing processes after the laser treatment and the other types of wound healing, such as burns or cuts, is the absence of myofibroblasts (specialized differentiated α-smooth muscle actin-positive DFs), which have the ability to shrink collagen fibrils and increase the formation of type I collagen [87]. This difference is associated with the relatively superficial and controlled nature of the thermal damage caused by the CO_2_ laser.

The third (reepithelization) phase of skin recovery after the thermal damage is the phase of complete healing [85,88]. During this phase, excess collagen is eliminated, in particular, by means of metalloproteinase MMP-13, which is involved in the remodeling of the newly formed collagen, whose level is increased at the final stage of healing, and the inflammatory cells are removed [85,88,89]. The regeneration of the epidermis occurs due to the increased proliferation/differentiation of living BKs in the ablation zone under the influence of growth factors produced by DFs.

The therapy using the ablative CO_2_ laser is characterized by high efficiency. However, the laser treatment technique is generally associated with a significant recovery period and may cause potentially serious complications, such as scarring, infections, and dyspigmentation. This limits the use of laser methods in cosmetological practice [84].

In this connection, the most promising innovative technique, called fractional laser photothermolysis, was proposed by Manstein et al. (2004) [90]. The fractional treatment is achieved through a pattern of microscopic thermal zones produced by a laser beam to a specific depth in the dermis [85]. Unlike the traditional ablative CO_2_ laser that damages the entire area of the skin subjected to the correction, the fractional laser (FL) affects only a fraction of the skin and mainly has a vertically oriented cylinder shape consisting of the thermally damaged tissue [90]. Each of these fractions is surrounded by unaffected skin that serves as a reservoir for stem/progenitor cells migrating to the damaged zone, providing the fastest healing of the damaged tissues [90,91]. The correction/treatment of skin defects using FL is characterized by rapid rehabilitation and has a minimal risk of side effects and complications.

Currently, both ablative and non-ablative FL devices have been developed and used. With both variants of treatment, the dermis is restored analogously to the physiological mechanism of wound healing that ends with the remodeling of the collagen and the collagen matrix [91,92,93,94,95,96]. Studies have shown that the use of FL devices for the purpose of collagen remodeling is characterized by high efficiency; the process of collagen production and the remodeling of the collagen matrix in the skin is observed from the first week after FL exposure and continues for 3 months [94,97,98].

##### PRP Therapy in Combination with Laser Technologies

A noticeable remodeling effect can be achieved by using FL in combination with autological platelet-rich plasma (PRP) [99,100,101]. PRP is a rich source of growth factors (GFs), cytokines, chemokines, and plasma proteins (fibrinogen, vitronectin, fibronectin, and thrombospondin) that are deposited in platelet α-granules, electron-dense corpuscles, and lysosomes [102,103]. Currently, it has been revealed that above 30 GFs are mainly contained in the alpha-granules of platelets, including the growth factors that are of particular interest to the skin cosmetic anti-age therapy, namely, vascular endothelial growth factor (VEGF), platelet-derived growth factor (PDGF), epidermal growth factor (EGF), fibroblast growth factor β (β-FGF)), transforming growth factor β1 (TGF-β1), and insulin-like growth factor (IGF). This set of GFs plays a key role in the regulation of tissue regeneration processes [102,103,104,105]. Following platelet activation, GFs are released from α-granules into the extracellular space by means of exocytosis. During the first 10 min, platelets produce about 70% of GFs, which are almost completely released within an hour. The additional amount of GFs is produced for at least 7 days [106].

The mechanism of the effect of PRP after skin laser therapy is associated with the paracrine effect of GFs/cytokines produced by platelets and is based on the molecular and cellular induction of the natural wound healing process [102,103,104,105,106,107,108]. The set of GFs contained in PRP promotes the chemotaxis of skin cells to the site of injury, as well as stimulates the following processes in the skin: proliferation and differentiation of skin cells, immunomodulation, angiogenesis, the synthesis of ECM components, and the remodeling of skin tissues [109]. In addition, since these two methods (laser and PRP therapy) are based on different biological mechanisms acting on the skin, it is reasonable to believe that their combined effect can be synergistic [110]. To date, many devices have been made available to obtain PRP [111], which can be applied both locally and through intradermal injections following the FL technique [112].

Thus, the considered methods used in the first stage of the cosmetological anti-age program allow one to renew the epidermis and remodel the dermal collagen; therefore, they improve the state and integrity of the collagen matrix, which is a prerequisite for the functioning of both resident and autological DFs used in the second stage of this program.

#### 4.1.2. The Second Stage 

The objective of the second stage of the program is to replenish the number of DFs that have decreased with age to maintain their activity. To solve the problem, the use of cultivated autological dermal fibroblasts (autoDFs), in our opinion, is the most efficient technique. To date, technology based on the application of cultivated autoDFs to correct age-related skin changes and scarring has been administratively recognized by aesthetic medicine in different countries. Thus, in Russia, “the SPRS^®^ therapy” has been used at the Human Stem Cells Institute (HSCI, OJSC, Moscow) since 2010 (Regulatory certificate issued by the Federal Service of Health Authorities on Russia # 2009/308 (21.08.2010). Medical technology “Intake, transportation, isolation, cultivation, cryopreservation, storage and application of autologous fibroblasts for correction of age-related and cicatricial changings of skin”).

Technology based on cultivated autoDFs has also been applied in other countries, including South Korea (“CureSkin”, S. Biomedics, since 2010; and “Rosmir”, Tego Sciences, since 2017), the USA (LAVIV^TM^ [azficel-T], Fibrocell, formerly Isolagen, since 2011), and Iran (“Renudermcell”, Celltech Pharmed, since 2018) [113].

This technology is based on autoDFs isolated from a biopsy sample taken from the patient’s skin (behind the ear) and introduced into the dermis through injections. AutoDFs are cultivated under laboratory conditions according to GMP standards and are then delivered to a cosmetological clinic for the procedure. The technology allows one to distribute autoDFs evenly with the appropriate density over the entire skin area that requires correction [114,115,116,117].

Our studies have shown (Table 2) that the immunophenotype of autoDFs is characterized by high expression levels of elastin and collagen of types I and III and the presence of markers CD73+, CD90+, and CD105+, confirming the mesenchymal origin of the autoDFs used [114]. The absence of hematopoietic markers (CD34− and CD45−) and epithelial markers (cytokeratins 14, 15, 16, and 19) has also been revealed. Additionally, we did not detect various protein products of differentiation markers except variable alpha smooth muscle actin expression under normal non-differentiational induced conditions. The quantitative analysis of proteins expressed in autoDFs (elastin, collagen of types I and III) revealed no statistically significant correlations between the age and sex of the patients (*p* > 0.05).

The safety of the autoDF therapy and the ability of autoDFs to effectively correct age-related skin changes were first demonstrated by Isologen (now Fibrocell Science Inc., Exton, PA, USA) in 1995 [115,116] and then confirmed through randomized multicenter placebo-controlled clinical trials [117]. In 2010, our histological studies were conducted at the Human Stem Cells Institute (Moscow, Russia) in the course of clinical studies (non-randomized, prospective, open-label, single-group studies with comparison to baseline facial skin status, 1 year observation period) [114]. The obtained results have demonstrated that one month following SPRS therapy, autoDFs were detected in the dermis as groups of cells without signs of mitotic activity. In the area of autoDF localization, a significant number of newly synthesized ECM elements was observed, whereas in the zones of transplantation, autoDFs were registered during the entire follow-up period, i.e., after 1, 3, 6, and 12 months. The occurrence of young (argyrophilic) collagen fibers in the form of thin convoluted black threads located inside fibroblast clusters was observed in all cases. This may indicate that the synthetic activity of autoDFs injected into the skin persists for at least a year.

Thus, there is no doubt that after the introduction of autoDFs into the dermis, they survive and are completely able to function, integrating with resident DFs and replenishing their pool. As a result, an increase in the number of collagen fibers was observed in the skin, while the thickness of the dermis increased by an average of 63% during the year, and the elasticity of the skin also increased [114]. Thus, the use of autoDFs allows one not only to increase the number of synthetically active DFs but also to remodel the dermal ECM through the de novo synthesis of collagen and other ECM components [114,115,116,117]. This is clinically manifested by an increase in skin density and a decrease in the number and depth of wrinkles. The effect gradually increases for six months, and then it reaches a plateau that can persist for at least 2 years. 

The second stage is conducted after 3 months following the end of the first stage since it takes up to 3 months for collagen remodeling after laser exposure [94,95,96,97,98].

#### 4.1.3. The Third Stage

The task of the third stage is to stimulate the biosynthetic activity of DFs in order to maintain the clinical result achieved in the previous two stages of the proposed anti-age facial skin correction program. The biological process of skin aging affects all skin structures and layers and is determined by various factors, such as genetic, epigenetic, endogenous, and exogenous factors. The exogenous factors primarily include UV light exposure [50,51,53]. Despite the fact that clinically significant results are achieved in the two previous stages of the program, it should be taken into account that the synthetic activity of autoDFs begins to decrease after 8–12 months following the procedure, whereas the aging factors continue to act. It means that to maintain the achieved clinical effect at a high level for a long time, it would be reasonable to periodically (depending on the individual characteristics of the patient’s skin) carry out the procedures promoting the activation of DFs. From our point of view, the optimal method of stimulating DFs is PRP therapy, whose mechanisms were described above. It has been shown that after PRP injections, the GFs contained in the α-granules of platelets bind to the corresponding transmembrane receptors on the surface of DFs and stimulate their synthetic activity [102,103,104,105].

### 4.2. Approximate Step-by-Step Scheme of Anti-Age Correction of the Facial Skin Using a Combination of Regenerative Methods

Stage I

1. Application of the fractional laser photothermolysis (FL): one to three procedures with intervals of 3–4 weeks, depending on the characteristics of the patient’s skin and the used FL type (ablative or non-ablative).
2. Conducting the PRP therapy of the skin: one procedure immediately after each FL procedure;
  (a) Intradermal PRP injections in the area exposed to FL after the non-ablative FL procedure;
  (b) Intradermal PRP injections or local PRP application throughout the area exposed to FL after the ablative FL procedure.

Stage II (3 months after the end of stage I)

A single procedure of the SPRS skin therapy is conducted to introduce the autoDF suspension (120 × 10^6^ autoDFs in a concentration of 15 × 10^6^ cells in 1 mL of saline solution) into the papillary layer of the patient’s face skin. The injections are performed using the retrograde linear technique using 30 G needles that are 13 mm in size [114].

Stage III (skin maintenance therapy, 8–12 months after stage II)

The PRP skin therapy is conducted through intradermal PRP injections; the number of procedures varies from one to three (depending on the individual characteristics of the patient’s skin), with intervals of 3–4 weeks.

**Figure 4:**
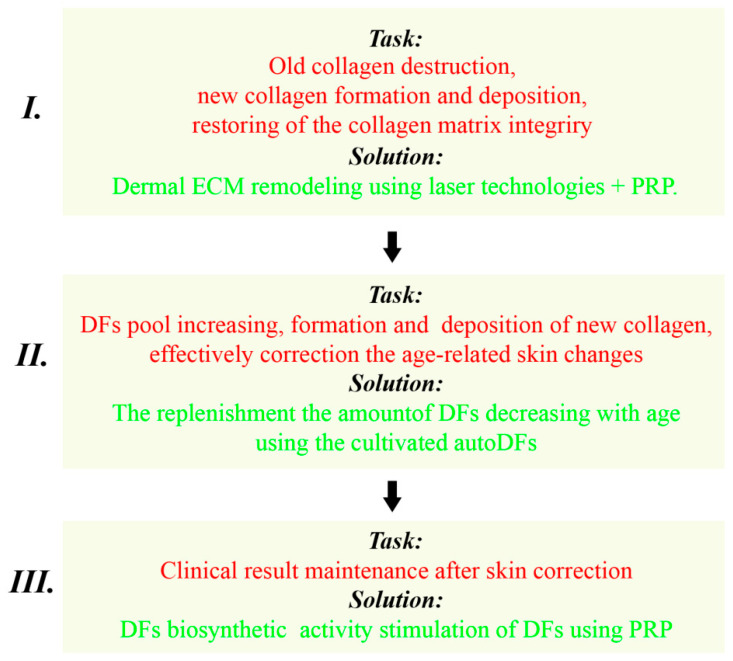
Stages of anti-age correction of the facial skin (the first stage of the program are to remodel the ECM of the dermis, the second stage is to replenish the number of DFs, the third stage is to stimulate the biosynthetic activity of DFs).

Our solution can be summarized by the presented scheme (Figure 4).

This scheme has been successfully used for 6 or more years in aesthetic medicine clinics in Russia to correct signs of chronological skin aging and photo-aging in patients of different ages and gender. A subjective assessment by both attending physicians and patients of the results of the application of the presented scheme confirms our conclusions: a significant improvement in the microstructure of the skin, an increase in its tension/elasticity, and a decrease in the number and depth of wrinkles. (The data relating to objective observation methods are not currently published).

## 5. Conclusions

The proposed program of anti-age skin correction using a combination of laser and cellular methods of regenerative medicine is aimed at stimulating skin renewal by affecting the age-related pathophysiological processes occurring at the molecular and cellular levels. The main target is dermal fibroblasts, as they are the general link that connects the skin’s biological processes. The program includes three stages, with the tasks and methods of their solution clearly defined for each stage. Thus, laser technologies make it possible to remodel the dermal collagen matrix and create favorable conditions for DF functioning, while cultivated autoDF therapy allows one to replenish the DF pool that decreases with age, and PRP therapy enables maintaining the achieved results by activating DF functions (if necessary). Each of the considered methods can be effectively used as a monotherapy. However, the consecutive (step-by-step) application of these methods enables enhancing the effect of the therapy on the molecular and cellular age-related processes, as well as stimulating the regeneration of skin tissues and, therefore, optimizing and prolonging the clinical effect of skin rejuvenation.

## Figures and Tables

**Table 1:** Types of dermal ECM collagens and their localization.

Collagen Types	Localization
Fiber-forming (fibrillar) collagens	
Large fiber-forming collagens	
Type I	Reticular dermis layer predominantly
Type III	Papillary dermis layer predominantly
Small (minor) fiber-forming collagens	
Type V	Both layers of the dermis
Non-fibrillar collagens	
FACIT collagens	
Type XII	Papillary dermis layer
Type XIV	Reticular dermis layer
Type XVI	Papillary dermis layer
Type XXII	Papillary dermis layer
Collagens forming network-like structures	
Basement membrane collagens	
Type IV	Basement membrane
Collagens of anchoring fibrils	
Type VII	Fibrils at the border of dermis and epidermis
Collagens of microfibrils	
Type VI	Microfibrils in both layers of the dermis
Transmembrane collagens	
Type XIII	Cellular membranes

**Table 2:** Protein autoDFs profiling based on own data.

Protein Profiling	
**Mesenchymal markers/expression-cells+%**	
CD73 (*NT5E* 5′-nucleotidase ecto)	Hi/>95
CD90 (*THY1* Thy-1 cell surface antigen)	Hi/>99
CD105 (*ENG* endoglin)	Hi/>98
Collagen I (*COL1A1* collagen type I alpha 1 chain)	Hi/>95
Collagen III (*COL3A1* collagen type III alpha 1 chain)	Hi/>95
Elastin (*ELN* elastin)	Hi/>95
Vimentin (*VIM* vimentin)	Hi/>98
Prolyl 4-hydroxylase (*P4HB* prolyl 4-hydroxylase subunit beta)	Hi/>99
**Endothelial and hematopoietic markers/expression-cells+%**	
CD31 (*PECAM1* platelet and endothelial cell adhesion molecule 1)	Low/<1
CD34 (*CD34* CD34 molecule)	Low/<1
CD45 (*PTPRC* protein tyrosine phosphatase receptor type C)	Low/<1
**Epithelial markers/expression-cells+%**	
CD324 (*CDH1* Cadherin 1, Type 1, E-Cadherin (Epithelial))	Low/<0.5
pan-Cytokeratin 14,15,16,19 (*KRT14*, *KRT15*, *KRT16*, *KRT19*)	Low/<0.5
**Differentiation markers/expression-cells+%**	
Aggrecan (*ACAN* aggrecan)—chondrogenic	Low/<1
Osteocalcin (*BGLAP* bone gamma-carboxyglutamate protein)—osteogenic	Low/<1
FABP4 (*FABP4* fatty acid binding protein 4)—adipogenic	Low/<0.5
a-SMA (*ACTA2* actin alpha 2)—smooth muscle	Variable/1–30
a-sk-Actin (*ACTA1* Actin Alpha 1)—skeletal muscle	Low/<1.5
sk-Myosin (*MYH1* Myosin Heavy Chain 1)—skeletal muscle	Low/<0.5
Myogenin (*MYOG* myogenin)—myogenic	Low/<0.5
MyoD1 (*MYOD1* myogenic differentiation 1)—myogenic	Low/<0.5

## Data Availability

Not applicable.
